# Recruitment using mobile telephones in an Irish general population sexual health survey: challenges and practical solutions

**DOI:** 10.1186/1471-2288-12-45

**Published:** 2012-04-04

**Authors:** Orla McBride, Karen Morgan, Hannah McGee

**Affiliations:** 1 Department of Psychology, Division of Population Health Sciences, Royal College of Surgeons in Ireland, 123 St. Stephen's Green, Dublin 2, Ireland

**Keywords:** Mobile telephones, Surveys, Methodology, Health, Sampling

## Abstract

**Background:**

Non-coverage of households without a landline telephone is a major concern of telephone survey researchers. Sampling mobile telephone users in national surveys is vital in order to gain access to the growing proportion of households that use mobile telephones extensively or exclusively. The complex logistics of conducting surveys with mobile telephones have been discussed in the literature. This paper outlines the actual challenges encountered during a recent national sexual health survey in Ireland, which utilized a mobile telephone sampling frame to recruit approximately half of the sample.

**Method:**

The 2010 Irish Contraception and Crisis Pregnancy Survey (ICCP-2010) is a nationally representative sample of adults aged 18-45 years living in Ireland (n = 3002; 1416 recruited by landline telephone and 1586 recruited by mobile telephone). The overall response rate for the survey was 69% (79% for the landline telephone strand; 61% for the mobile telephone strand). All interviews were conducted using computer-assisting telephone interviewing.

**Results:**

During the 18-week fieldwork period, five main challenges relating to the use of mobile telephones were encountered: (1) explaining to respondents how random digit dialling works in relation to mobile telephones; (2) establishing the respondent's eligibility; (3) calling the respondent with the Caller ID blocked or withheld; (4) calling the respondent when they are in any number of locations or situations; and (5) explaining to respondents the importance of refusal conversion calls for the response rate calculation. Details of how the survey protocols and procedures were monitored and adapted throughout the study to ensure a high response rate are outlined.

**Conclusion:**

It is undeniably more challenging to recruit respondents using mobile telephones as opposed to landline telephones. Respondents are generally not familiar with being contacted on their personal mobile telephone for the purposes of being recruited for a research study. The main challenge for survey methodologists and interviewers is to devise simple protocols to explain to respondents why they are being contacted on a mobile telephone. Recommendations for survey researchers interested in using this methodological approach in the future are discussed.

## Background

Telephone surveys have played a vital role in public health research and practice for many decades [1,2]. Telephone interviews have a number of important advantages over face-to-face surveys: (1) they are usually more cost and time effective; (2) the sampling frame can include areas that may be hard for interviewers to access in person; and (3) respondents have a sense of anonymity once they understand that their telephone number was chosen at random, which can increase the validity of information being reported [3]. In recent years, several knowledge, attitudes and beliefs (KAB) telephone surveys have been conducted successfully in Ireland, for example, the Sexual Abuse and Violence in Ireland Study (SAVI-2001)[4], the Irish Contraception and Crisis Pregnancy Study (ICCP-2003) [5] and the Irish Study of Sexual Health and Relationships (ISSHR-2006) [6].

Traditionally, sampling frames for telephone public health surveys in Ireland and elsewhere have been limited to landline telephone numbers [7]. Recent research in the US, Australia, and Europe has demonstrated that the percentage of the population who do not have a landline telephone in their home has steadily decreased in the last few decades [1,8-11]. For example, it is estimated that approximately 23% of US households in 2009 were mobile telephone only households, an increase from 2% in 2003 [12]. In Ireland, a recent national face-to-face household survey of lifestyles, attitudes and nutrition (SLÁN 2007) [13] revealed that 61% of respondents had access to a mobile and landline telephone where they lived; however, a substantial percentage (23%) of individuals only had access to a mobile telephone [13]. Extensive research in the US has revealed that households using mobile telephones exclusively are disproportionally younger, male, single, and living in rental accommodation when compared to households that have a landline telephone [7,14]. This population of adults leading a 'wireless only' lifestyle [11] are also more likely to have higher rates of detrimental health behaviours compared to adults who live in households that use a landline telephone [15].

Non-coverage of households without a landline telephone is a major concern of telephone survey researchers [7,9]. It is clear that sampling mobile phone users in national surveys is vital to gain access to the growing proportion of households that use mobile telephones extensively or exclusively [16]. Recently, the feasibility of recruiting respondents via mobile telephones for national health surveys, such as the 2007 California Health Interview Survey [17] and the Behavioural Risk Factor Surveillance System (BRFSS) [18], has been investigated. As a result of these and other methodologically pioneering studies, the challenges associated with conducting research using mobile telephones have been debated within the literature [1,17]. These challenges include, but are not limited to, the following: (1) safety: the respondent could receive a mobile telephone call in any location (e.g. whilst driving a car) (2) privacy: the respondent could be responding to the survey in a location where they may not be able to comfortably disclose sensitive information or they could feel that an unsolicited call on their personal mobile telephone is an invasion of their privacy, unlike the typical private household landline situation of traditional surveys; (3) eligibility: it can be difficult to determine whether the mobile phone owner is an adult and/or eligible for a study; (4) cost for the respondent: the respondent may incur a charge if they are accessing a mobile network other than their service provide ('roaming') or on a pre-paid plan that charges for incoming calls (often referred to as 'Receiver Party Pays'- commonly used in countries such as the US, Canada, and China) [19]; (5) cost for the commissioning agent: the cost of calling mobile telephones to recruit respondents is more expensive compared to landline telephones; (6) legality: calling private mobile telephones may breach communication legislation (e.g. the US 1991 Telephone Consumer Protection Act); (7) identification: it is not as easy to identify whether the mobile phone is being used for business or personal matters; (8) connections problems: once a connection has been established, it is possible for the call to be 'dropped' due to lack of mobile network coverage or battery supply; (9) respondent burden: given that people are often under special time constraints and special pressures when speaking on mobile telephones, surveys administered via a mobile telephone may need to be shorter than those conducted via landline telephones [20]; and (10) singular nature of mobile telephone: a landline telephone is normally used by several members of a household, whereas a mobile telephone has generally one primary user. In surveys, if a respondent answers a landline telephone and is either ineligible or unwilling to complete a survey, an interviewer can prompt for another eligible individual in the household to complete the survey; this is not possible using mobile phones.

All of the issues outlined above have the potential to seriously impede on the success of surveys that utilize mobile telephones to recruit respondents. For example, it has been suggested that response rates to telephone surveys in Ireland, the UK, and elsewhere have generally diminished over recent years because householders are often inundated by requests from market research companies (MRC) to participate in telephone surveys [8]. Using mobile telephones to recruit respondents has considerably reduced the response rate of national surveys in the US [11]. It is vital, therefore, that considerable efforts are made to try and overcome these obstacles.

The aim of this paper was to document the challenges encountered during a recent Irish KAB survey called the 2010 Irish Contraception and Crisis Pregnancy Survey (ICCP-2010). ICCP-2010 is a cross-sectional, nationally representative telephone survey of knowledge, attitudes and behaviours in relation to crisis pregnancy, contraception and sexual health, among adults aged 18-45 years living in Ireland (*n *= 3002). The survey is unique for two major reasons: (1) it was the first national survey in Ireland to split the recruitment for the survey over mobile and landline telephones, which enhances the representativeness of the sample; and (2) respondents were not incentivised to participate but a high overall response rate of 69% was achieved, which is unusual for surveys using mobile telephone recruitment [1]. The aim of this paper is to outline how the ICCP-2010 was conducted, with a particular focus on describing the main challenges encountered while recruiting a large random sample using mobile telephones and the outlining the practical steps taken to overcome these challenges successfully to achieve a high response rate from a nationally representative sample of the general population.

## Methods

### Context

The ICCP-2010 was funded by the Health Service Executive Crisis Pregnancy Programme (CPP), formerly the Crisis Pregnancy Agency (CPA). The fieldwork for ICCP-2010 was conducted by a MRC between August and November 2010. Ethical approval for the study was obtained from the Research Ethics Committee of the Royal College of Surgeons in Ireland (RCSI). As previously mentioned, a similar survey was conducted in 2003 (ICCP-2003). The main aim of the ICCP-2010 was to capture information pertaining to new phenomena and emerging trends in the domain of sexual health and pregnancy over the seven year period between the two surveys. The major difference between the two surveys is that the ICCP-2003 only recruited respondents using landline telephones whereas ICCP-2010 utilized both mobile and landline telephones.

### Interviewing team

The interviewing team consisted of approximately 25 women, all of whom had a great deal of experience administering questionnaires using computer assisted telephone interviewing (CATI). All interviewers participated in a comprehensive two-day training session, which was administered by the project team.

### Questionnaire & timing

The questionnaire for this survey was similar to the one administered in ICCP-2003 to allow for comparison between the two surveys. The questionnaire for ICCP-2010 was refined slightly through discussion with an Advisory Group, consisting of professionals from the pharmaceutical industry, the Equality Authority in Ireland, and the primary care sector. One major concern prior to commencing the main fieldwork for the survey was ensuring that the questionnaire would be short enough to be administered via a mobile telephone. Based on a pilot survey of 192 interviews, it was estimated that the average administration time for the survey would be 21 minutes, 49 seconds (range 5 minutes 18 seconds to 44 minutes 9 seconds). The average time was very similar across the mobile and landline telephone strands. In the main study, once a respondent agreed to participate in the survey, the interview was often completed within a single setting. There was no notable negative feedback from respondents or the interviewers in relation to the duration of the interview.

### Interviewing software

The ICCP-2010 questionnaire was administered using a CATI system called NIPO software [21]. NIPO software manages different research methodologies, including computer-assisted personal or web interviewing (CAPI; CAWI) and CATI, and can also serve as a data entry package. It also manages the telephone numbers for the survey, whereby landline and mobile telephone numbers are automatically sent to interviewers for manual dialling. NIPO software is capable of managing appointments with respondents at a specific date and time, and when that appointment is due, the number is extracted from the telephone database and will be dialed by an interviewer on duty. If for some reason a telephone number is not answered when it is dialed (e.g., it is not answered or ringing busy), the software records this information and reschedules this number to be dialed again on a different day/time.

### Sampling frame for landline telephones

All telephone numbers utilised in this survey were generated using random digit dialing (RDD). A three-stage clustered sampling design was used for sampling landline telephone numbers. The first stage involved the selection of the primary sampling unit (PSU) from the GeoDirectory which is a listing of all residential addresses in Ireland. The GeoDirectory does not contain a corresponding telephone number for each residence. Using this information, sampling points are based on aggregates of townlands, with a minimum of 500 residential addresses in each one. The PSUs are selected at random by systematic selection following a random start, having being sorted by characteristics of the area. At the second stage, an address was randomly selected within the PSU. The address is used to look up a telephone number stem. The 'hundreds bank' method was then used to change the last two digits of the stem to create a full set of 100 numbers ranging from "XXXXX00" to "XXXXX99". This procedure means that telephone numbers that are listed in the national telephone directory as well as those that have been excluded or 'unlisted' were generated. Many of the generated telephone numbers were not relevant for the study; for example, some telephone numbers were businesses or other non-residential locations. This meant that a lot of numbers in each PSU had to be screened out, but this process does not adversely impact on the statistical nature of the resultant sample. The third stage is the selection of the actual individual within the household who will complete the questionnaire. We imposed a post-stratification selection rule in the selection of the individual within the household chosen for interview. This quota sampling technique was derived from estimates obtained from the 2010 Quarterly National Household Survey [22]. This procedure ensures a representative mix of men and women in different age bands from different regions throughout the country (see Table 1). Without this post-stratification selection criterion, some categories (e.g. older women) would be over-represented in the final sample for analysis, as these groups are easier to reach and tend to have a higher response rate. The quotas achieved for the survey overall were between +/- 2% of the population (see Table 1). This provides support that the sample obtained was representative to the general population in relation to sex, age, and region.

**Table 1:** Estimated and achieved quotas for the 2010 Irish Contraception and Crisis Pregnancy Survey (ICCP-2010)

Categories		**Estimated**^**1**^		Achieved		Difference between sample and population
			
		Sample size (*n *= 3000)	Percentage of population	Sample size (n = 3002)	Percentage of population	
Region	Dublin	930	31%	947	31%	0%

	Rest of Leinster	780	28%	816	26%	1%

	Munster	795	25%	757	27%	-2%

	Connacht and Ulster	495	16%	482	17%	-1%

Men by age	Men 18-25 years	315	9%	285	11%	-1.5%

	Men 26-35 years	615	18%	571	21%	-2%

	Men 36-45 years	570	20%	586	19%	0.5%

Women by age	Women 18-25 years	315	11%	331	11%	0%

	Women 26-35 years	615	22%	638	21%	0%

	Women 36-45 years	570	20%	591	19%	1%

Note. ^1 ^Quotas for the survey were derived from the 2010 Quarterly National Household Survey.

### Sampling frame for mobile telephones

Currently, there is no publically available database of mobile telephone numbers in Ireland. All Irish mobile telephone operators automatically place all their customers on the National Directory Database Opt-Out Register. This process ensures that their subscribers are not subjected to direct marketing calls. As a result, the volume of publically available mobile telephone numbers is very low. The MRC conducting the fieldwork for this study had to devise a method of providing a large database of mobile telephone numbers for this survey. Over the last few years, the MRC had established a large file of 'real' mobile telephone numbers that were used for other research studies. These real mobile telephone numbers were used as the 'stems' for the mobile database. Similar to the landline telephone approach, the last two digits of the 'real' mobile telephone numbers were substituted with the digits 00 to 99. This resulted in 100 mobile telephone numbers being generated. The 'real' mobile telephone numbers were omitted from the database in accordance with Irish Data Protection Legislation. The creation of these new numbers means a proportion of the database were likely to be ineligible either being businesses or inactive telephone numbers, but the process ensured that both listed and unlisted numbers will be covered. There are five mobile telephone number prefixes in Ireland operated by different service providers (083: Three; 085: Meteor; 086: O2; 087: Vodafone, 088: Digiweb, and 089: Tesco). While mobile numbers are portable between operators, all new numbers are issued in an operators own allocation. Unlike in some other countries, for example the US [11], mobile telephone number prefixes in Ireland do not correspond to specific regions of the country. The substantial database of mobile telephone numbers generated for this study, therefore, contained mobile telephone numbers from all network providers that were distributed throughout the country.

### Selection of an individual to complete the survey

As outline above, estimates from the 2010 Quarterly National Household Survey [22] were used to derive sex, age, and region quotas for this survey's sample. In terms of selecting an individual to complete the survey, when all quotas were available, any eligible respondent answering a landline telephone within a household was permitted to complete the survey. When specific quotas were filled, the interviewer asked to speak to another respondent within the household to fill the remaining quotas. For mobile telephones, only the owner of the mobile telephone, or the person who answered the mobile telephone (if not the owner), was permitted to complete the survey if they were eligible. Once quotas were filled on the mobile telephone strand, additional telephone numbers were dialed until respondents willing the vacant quota requirements were recruited.

### Procedure for calling mobile and landline telephones in the survey

The CATI centre was open from 9 am-9:30 pm Monday to Friday, and from 9 am-4 pm on Saturday. All telephone numbers were manually dialled by the interviewing team from a system using a blocked Caller ID. If the call is not answered, it will register as a 'missed' call from a 'private' or 'unknown' number on Irish landline and mobile telephones. No voicemails or text messages were sent to the respondent to inform them about the purpose of the survey. The reason for this practice was two-fold: (1) the interviewing team can contact thousands of people per day across different surveys and it would be extremely difficult to manually manage all of the incoming calls if respondents were able to call back into the CATI centre having received a 'missed' call on their telephone; and (2) it prevents the respondent from having to incur a cost by calling into the CATI centre to return the 'missed' call they received. The NIPO software manages the randomly generated telephone numbers so that the interviewing team can contact the respondent again at another time. All telephone numbers could be dialled up to a total of ten times, on different days and at various times throughout the day, over a three-week period. This means that an interviewer(s) could speak to an individual or individuals within a household, on a number of different occasions in an attempt to schedule an appointment to conduct the survey with an eligible adult. Any telephone number that was ringing out, ringing busy, or diverted to voicemail, was automatically reloaded into the CATI system to be dialled at a different time over the three-week period.

When the telephone call was answered, the interviewer introduced the nature and purpose of the survey to any adult who answered the telephone. Obviously, if a child answered a landline or a mobile telephone, the interviewer asked to speak to a parent or guardian. Many children under the age of 18 years of age own a personal mobile telephone [11]. Given that cold calling mobile telephones is a relatively new recruitment technique in Ireland, coupled with sensitive nature of the study, it was decided at the beginning of the survey to ask all respondents answering a mobile telephone if they were over the age of 18 years. This practice is consistent with the Market Research Society Code of Conduct [23]. If the respondent was over the age of 18 years, the purpose and nature of the survey was explained and the respondent was invited to participate in the survey. If the respondent was under the age of 18 years, the interviewer asked to speak to a parent or guardian to explain why their child's personal telephone had been called using RDD.

To obtain an accurate response rate for both telephone strands, the interviewer ascertained whether the adult would be willing to participate in the survey, before determining if they met the eligibility criteria for the study (i.e. between 18-45 years of age).

All respondents who were invited to participate in the study were coded as follows:

1. The respondent was eligible and completed interview

2. The respondent agreed to complete the survey, but not at the time of contact (i.e. *non-refusal*)

3. The respondent was non-committal about participating in the survey (i.e. *non-refusal*)

4. The respondent refused to do the survey (i.e. *soft refusal*)

5. The respondent refused to do the survey and asked not to be contacted again (i.e. *hard refusal*)

Respondents categorized into codes 1 or 5 were not contacted again by the interviewing team.

The eligibility of all respondents categorized into codes 2-4 had to be established. This involved asking the respondent their exact age to establish if they were in the required age group (i.e., 18-45 years). It is not always possible to establish eligibility on first contact. Telephone numbers of respondents categorized into code 2 were re-entered into the system to be reloaded at either a specific time outlined by the respondent, or at a day and time that was randomly generated by the CATI system if a definite date was not given by the respondent. Telephone numbers of respondents categorized into code 3 are re-entered into the system to be dialled at a randomly generated day and time, within three days of the initial contact. Telephone numbers of respondents categorized into code 4 were re-contacted again after a three-week period had passed (up to a total of 5 contacts), to try and reach the respondent one final time to invite them to re-consider their decision to take part. This process of 'refusal conversion' is standard international practice in telephone surveys [24]. For respondents categorised into codes 2 and 3, the interviewing team contacted the potential respondent up to a total of 10 times following verbal contact, to try and obtain a successful interview, or to establish eligibility.

If a respondent's eligibility could not be established following 10 contact attempts over a three-week period, their eligibility was estimated as a percentage (8%) of those respondents for whom eligibility could be established, consistent with guidelines from the American Association for Public Opinion Research [25].

## Results and discussion

### Total interviews and response rates

**Figure 1:**
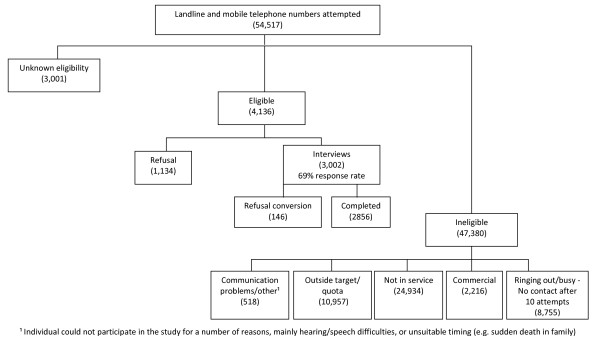
**Profile of unique phone numbers called and outcome classification for survey**.

A breakdown of each telephone number dialled (landline and mobile telephone numbers combined) is presented in Figure 1. The overall response rate for the survey was 69% (79% for landline telephones; 61% for mobile telephones). It was necessary to dial a larger number of mobile telephone numbers to fulfil the target quotas for the survey. This means that a slightly larger percentage of the survey was completed via mobile telephone (52.8%) compared to landline telephone (47.2%). Table 2 provides information relating to the sample recruited through the landline and mobile telephone strands. More men, young adults, and single respondents were recruited via the mobile telephone strand as opposed to the landline telephone strand.

**Table 2:** Demographic characteristics of the ICCP-2010 sample, by telephone type

Demographic characteristics	Landline (n = 1,418)		Mobile (n = 1,584)	
	
	Un-weighted Sample (n)	Weighted overall sample (%)	Un-weighted sample (n)	Weighted overall sample (%)
**Sex**				

Men	520	13	920	37

Women	898	24	664	26

**Age**				

18-25	338	9	379	15

26-35	508	12	721	30

36-45	572	16	484	18

**Marital status**^**1**^				

Single/cohabiting	648	17	910	38

Married	714	19	610	23

Separated/divorced/widow	56	1	60	2

**Highest education**				

Pre-Leaving Certificate	150	6	208	11

Leaving Certificate	366	13	365	18

Post-Leaving Certificate	902	19	1,011	33

**Region**				

Dublin	413	10	531	20

Rest of Leinster	414	12	404	16

Munster	317	8	440	18

Connacht and Ulster	274	8	209	8

**Country of birth**				

Republic of Ireland	1,131	29	1,310	49

Northern Ireland	31	1	13	< 1

UK	105	2	83	3

Rest of EU	56	3	77	6

Africa	38	1	38	2

Asia	22	< 1	34	2

USA/Canada/Australia/New Zealand	21	< 1	14	1

Elsewhere	14	< 1	15	1

Note. ^1 ^Missing data on marital status for four respondents recruited via mobile telephone.

### Feedback from interviewing team and survey respondents

Information on how the protocols and procedures for the survey were perceived by the respondents was obtained from a number of sources: (1) regular meetings were held with the interviewing team to obtain information about how the survey was progressing, and whether the respondents had any comments on the content or nature of the survey; (2) respondents were offered details of a freephone telephone number which could be used to contact the project team to relay any feedback in relation to their experience; and (3) respondents could also contact the project team using contact details on the RCSI institutional website.

Six respondents in total (0.2% of total sample) wrote to the authors to document their experience with the survey. All of these respondents were contacted via mobile telephone. Considering the information obtained from these respondents in conjunction with more general feedback obtained from the interviewing team, several modifications were made to the original procedures and protocols during the fieldwork period for the survey. In the next section, the five main challenges encountered during the fieldwork period are outlined, as well as the action that was taken to resolve them.

### Challenges encountered during ICCP-2010

#### Challenge 1: Calling the respondent from a telephone system with a blocked Caller ID

As previously mentioned, all telephone numbers were dialled from a telephone system with a blocked Caller ID. It is widely recognised that the Caller ID facility on telephones is frequently used to screen calls, particularly by younger adults, those who have never been married, and those who live in more densely populated areas [11,26]. In the current study, the percentage of valid telephone numbers that went unanswered after 10 contact attempts was higher for the mobile telephone strand (30%) compared to the landline telephone strand (24%). This suggests that mobile telephone owners maybe more reluctant to answer with a blocked Caller ID. They may also become irritated at receiving 'missed' calls from an unknown source on their personal mobile telephone. Other respondents were also concerned about the validity of the survey, given that the call was being made from an unidentified telephone number.

#### Challenge 1: Action Taken

1. Interviewers were provided with additional information to explain to the respondents why it is necessary to dial from a telephone system using a blocked Caller ID at the CATI centre. If necessary, interviewers were able to offer the respondent a recall on either their landline or mobile telephone with the Caller ID unblocked, so that the respondent could have the contact details for the MRC. This could be used for validation purposes. A small number of respondents availed of this service and were then happy to complete the interview.

2. Interviewers were able to give respondents a link to the RCSI institutional website for additional verification for the study. Many respondents recorded this information and subsequently completed the survey.

3. Bearing in mind that singular nature of mobile telephone ownership compared to the household nature of a landline telephone, it was considered appropriate during the survey to reduce the number of contact attempts made to mobile telephone owners. Specifically, it was decided that contacting potential respondents on mobile telephones should cease upon (1) the number has been dialled eight times; or (2) the interviewer makes verbal contact with the respondent three times. This change in the procedure appeared to have a positive effect in reducing respondent burden and irritation.

#### Challenge 2: Explaining how RDD works, particularly in relation to mobile telephones

Many respondents queried how their telephone number had been obtained for the survey. Although this was a bigger issue for mobile telephone owners, some landline telephone owners were annoyed that their telephone number was dialled given that they excluded their telephone number from the national telephone directory. In relation to mobile telephones, some respondents were aware of the legislation that prevented Irish mobile telephone owners from receiving unsolicited calls on their mobile telephone, but were not aware that social or market research was not covered under this legislation.

#### Challenge 2: Action Taken

1. Interviewers were given an additional simplified script to explain how landline and mobile telephone numbers were generated using RDD. All respondents were reassured that their mobile telephone number had not been purchased from a database or other external source for this study. It was explained that the interviewer had no information about the owner of the telephone number.

2. In relation to the legislation, the following script, administered by the interviewers or the authors, appeared to alleviate some of the respondents' concerns: "There is legislation in place from the Data Protection Commissioners to enable consumers to prevent getting calls for marketing purposes. However, social or market research is not a direct marketing activity and is therefore not covered by this legislation. The following websites can be accessed for more information in relation to this matter: (1) the Commission for Communications Regulator (ComReg) [27] and (2) the Data Protection Commissioners [28]."

#### Challenge 3: Appropriateness of calling respondent when they are in any number of locations

As noted in the literature, one of the major challenges associated with conducting research via mobile telephones relates to safety and privacy. Some respondents were driving when they answered the telephone call and complained that it was not a good time for them to complete the survey. A few respondents were of the opinion that it was inappropriate to ask questions relating to sexual health via a mobile telephone survey.

#### Challenge 3: Action taken

1. Interviewers were instructed to be proactive and listen for clues as to whether the respondent might not be in a position to answer the survey and to offer the respondent a call-back at a more convenient time.

2. Interviewers offered reassurance to respondents that a similar sexual health survey had been conducted seven years earlier using a similar methodology and stressed the importance of the survey for developing sexual health and pregnancy-related services in Ireland.

#### Challenge 4: Establishing the eligibility of the respondent

One of the major difficulties encountered over the course of the survey was establishing the eligibility of the respondent, particularly on the mobile telephone strand. As previously outlined, the interviewer was required to establish whether the respondent was over 18 years of age before divulging information about the nature of the survey. Many respondents seemed to be irritated by this procedure. In total, 3.9% of all respondents contacted via mobile telephone would not confirm they were 18 years or older, even after the interviewer explained that this procedure was just to ensure that the interviewer was not speaking to a minor.

Once the respondent confirmed they were over 18 years, the nature and purpose of the survey was outlined and they were invited to take part. Regardless of whether they agreed to participate in the survey, the interviewer inquired about the respondent's age to assess eligibility. This process appeared to aggravate a lot of respondents. Many complained that they should not be required to report their age when they were not interested in participating in the survey.

#### Challenge 4: Action taken

1. Any respondent who would not confirm that they were not over the age of 18 years were coded as ineligible and were not contacted again by the interviewing team.

2. If respondents refused to take part in the study, but did not appear to be distressed or irritated by the contact, the interviewers were instructed to try and explain the importance of establishing the respondent's eligibility, as follows: "I appreciate you don't want to take part in the research; however, it is important for us to know how many people in the relevant age group have been asked, and how many take part and how many refuse, just so we can say how representative the study is. Do you mind as a last question if I ask your age for these reasons? ". Alternatively, the interviewer may have felt it was more appropriate to simply ascertain if the respondent is under or over 45 years of age (the upper age limit for the survey).

#### Challenge 5: Refusal conversion procedure

As previously outlined, all respondents who refused to participate in the survey upon first contact were re-contacted one final time to afford them an opportunity to re-consider their decision. Some respondents seemed to be annoyed that they were being re-contacted given that they had already declined to participate in the study. Some respondents felt that their privacy was being invaded because they were being contacted repeatedly when they believed they had declined to participate. For example, many respondents were non-committal about participating in the survey at the first contact but did not outright refuse to participate. These respondents were contacted again several days later to see if they would be interested in completing the interview. Some respondents were confused and irritated by this procedure because they believed that their non-committal response at the first contact was actually a refusal to complete the survey. However, given the procedure for this survey, these respondents were only coded as a 'soft refusal' at the second contact, and were therefore eligible for the refusal conversion procedure approximately three weeks later. In summary, a large percentage of respondents have spoken to members of the interviewing team approximately 3 times, and several respondents viewed this as an excessive invasion of their privacy.

#### Challenge 5: Action taken

1. Additional interviewer training was conducted to ensure that the interviewers were particularly focused on classifying the respondents correctly as a soft or hard refusal following the initial contact. Increasing interviewer accuracy in this procedure reduced the number of contact attempts that were made to the respondent.

2. The following script was given to interviewers to help them explain the importance of the refusal conversion calls: "Sometimes, people are unsure whether or not they want to be involved in a research study. If they want to reconsider their decision at a later point in time, they do not have any means of contacting the interview team. Considering this issue and the importance of representativeness, it is standard international practice to re-contact one time only those individuals who initially declined to participate in the study. This process of re-contacting individuals will not include any individual who specifically asked not to be re-contacted in relation to this study. However, we often find that people are often glad of the opportunity to take part, having had some time to think about their decision."

3. Approximately half way during the survey, the refusal conversion procedure for the mobile telephone strand was stopped, to eliminate any additional respondent burden. This meant that approximately 12% of mobile telephone respondents who were eligible to participate in the survey were not re-contacted and asked to re-consider their decision to participate. This methodological decision is likely to have impacted negatively on the response rate that was achieved for the mobile telephone compared to the landline telephone strand (61% vs. 79%). This change in procedure appeared to have the desired effect, however, in that no additional feedback from respondents in relation to privacy issues was received after this point in the survey.

## Conclusions

The ICCP-2010 was pioneering in a national and international context because it demonstrated that recruitment using mobile telephones in general population public health surveys is feasible and successful when survey protocols and procedures are closely monitored and revised throughout the fieldwork period. Effective call scheduling protocols are imperative for achieving a high response rate in telephone surveys [11] and this would seem particularly true for surveys using mobile telephones. Without incentivising respondents, the ICCP-2010 was able to receive much higher response rates than other surveys using mobile telephones in the US [11,16]. Whilst the response rate for the mobile telephone strand of the survey was lower than that of the landline telephone strand, the overall response rate was still very acceptable (61%). Generally, this response rate is consistent with European research that has suggested that mobile surveys do not usually produce response rates that much lower than fixed landline surveys [29].

Mobile telephone surveys reach a population not currently represented in landline RDD samples. The use of a mobile telephone strand in national surveys is vital for recruiting young adults, particularly young males, who are becoming increasingly difficult to contact. In ICCP-2010, it was necessary to conduct more interviews via the mobile telephone strand in order to fulfil the target quotas for this age group, and obtain a nationally representative sample. If this strand had not of been included, it is unlikely that the sample would have been a representative sample of 18-45 year old adults living in Ireland in 2010.

It is undeniable that it is more challenging to recruit respondents via mobile telephone as opposed to landline telephones. In our experience, this is largely because respondents are not familiar with being contacted on their personal mobile telephone, and are somewhat suspicious as to how their mobile telephone numbers have been obtained. The main challenge for survey methodologists and interviewers is to devise simple protocols to explain to respondents why they are being contacted on a mobile telephone. These protocols need to be modified or adapted to comply with feedback from respondents to reduce the degree of respondent burden. In conclusion, telephone survey approaches in the future will have to work with an increasingly mobile telephone culture and so it is essential that researchers take the opportunity to reflect on and report on strategies that enhance or detract from the success of such approaches.

## Competing interests

The authors declare that they have no competing interests.

## Authors' contributions

All authors were members of the project team for the 2010 Irish Contraception and Crisis Pregnancy Survey: HMG and KM were co-principal investigators and OMB was the project manager. All authors were involved in the development and revision of study protocols and procedures discussed in this paper, which ensured the survey was completed successful and obtained a high response rate. HMG and KM conceptualised the design of this paper. OMB drafted the entire manuscript. HMG and KM provided critical feedback on earlier drafts of this manuscript. All authors read and approved the final manuscript.

## Authors' information

All authors are attached to the Department of Psychology, Division of Population Health Sciences, Royal College of Surgeons in Ireland, 123 St. Stephen's Greene, Dublin 2, Ireland.

## Pre-publication history

The pre-publication history for this paper can be accessed here:

http://www.biomedcentral.com/1471-2288/12/45/prepub
